# Adiposity, inflammation, genetic variants and risk of post-menopausal breast cancer findings from a prospective-specimen-collection, retrospective-blinded-evaluation (PRoBE) design approach

**DOI:** 10.1186/2193-1801-2-638

**Published:** 2013-11-27

**Authors:** Xiaowei Sherry Yan, Jill Barnholtz-Sloan, Xin Chu, Ling Li, Ryan Colonie, Jessica Webster, Diane Smelser, Nikitaban Patel, Jeffery Prichard, Azadeh Stark

**Affiliations:** Center for Health Research, Geisinger Health System, Danville, PA 17822 USA; Case Comprehensive Cancer Center, Case Western Reserve University School of Medicine, Cleveland, OH 44106 USA; Weis Center for Research, Geisinger Health System, Danville, PA 17822 USA; Department of Pathology and Laboratory Medicine, Geisinger Health System, Danville, PA 17822 USA; Department of Pathology and Laboratory Medicine, Henry Ford Health System, Detroit, MI 48202 USA; Center for Clinical Epidemiology and Biostatistics, School of Medicine, University of Pennsylvania, Philadelphia, PA 19104-6021 USA

**Keywords:** Metabolic syndrome, Post-menopausal breast cancer, Chronic inflammation, MAP3K1

## Abstract

Chronic internal inflammation secondary to adiposity is a risk factor for sporadic breast cancer and Post-Menopausal Breast Cancer (PMBC) is largely defined as such. Adiposity is one of the clinical criteria for the diagnosis of Metabolic Syndrome (MetS) and is a risk factor for PMBC. We examined SNPs of eight genes implicated in adiposity, inflammation and cell proliferation in a Prospective-specimen-collection, Retrospective-Blinded-Evaluation (PRoBE) design approach. A total of 180 cases and 732 age-matched controls were identified from the MyCode prospective biobank database and then linked to the Clinical Decision Information System, an enterprise-wide data warehouse, to retrieve clinico-demographic data. Samples were analyzed in a core laboratory where the personnel were masked to their status. Results from multivariate logistic regression yielded one SNP (rs2922126) in the GHSR as protective against PMBC among homozygotes for the minor allele (A/A) (OR = 0.4, 95% CI 0.18-.89, P-value = .02); homozygosity for the minor allele (C/C) of the SNP (rs889312) of the gene MAP3K1 was associated with the risk of PMBC (OR = 2.41, 95% CI 1.25-4.63 P-value = .008). Advanced age was protective against PMBC (OR = 0.98, 95% CI 0.95-0.99, P-value = .02). Family history of breast cancer (OR = 2.22, 95% CI 1.14-4.43. P = .02), HRT (OR = 3.35; 95% CI 2.15-5.21, P < .001), and MetS (OR = 14.83, 95% CI 5.63-39.08, P < .001) and interaction between HRT and MetS (OR = 39.38, 95% CI 15.71-98.70, P < .001) were associated with the risk of PMBC. We did not detected significant interactions between SNPs or between the SNPs and the clinico-demographic risk factors. Our study further confirms that MetS increases the risk of PMBC and argues in favor of reducing exposure to HRT. Our findings are another confirmation that low penetrance genes involved in the inflammatory pathway, i.e. MAP3KI gene, may have a plausible causative role in PMBC. Given the fact that genetic constitutionality of individuals cannot be changed, efforts should be focused on life style modification.

## Background

Post-menopausal breast cancer (PMBC) is largely defined as a sporadic disease, as most women diagnosed with PMBC do not have a first degree family history of breast cancer. Of all identified modifiable risk factors for PMBC, adiposity has been known to have the strongest (Risk ratio = 1.67) and the largest population attributable risk (>20%) (Sprague et al. 2008). Adipose tissue as an endocrine organ is metabolically active and is involved in several biochemical pathways; the association between adiposity and PMBC most likely is not limited to one pathway or biochemical mechanism, per se (Galic et al. 2010). In post-menopausal women, peripheral adipose tissue is the primary source of circulating estrogens which are synthesized from its androgen precursors (Carmichael 2006; Gruber et al. 2002). Extensive epidemiologic and clinical correlative studies support that post-menopausal adiposity is associated with elevated circulating levels of estradiol and estrone and the risk of hormone positive breast cancer (Missmer et al. 2004; Cummings et al. 2009; McPherson et al. 2000). Furthermore, chronic internal inflammation secondary to adiposity has been associated with the risk of PMBC (Aghamohammadzaeh and Heagerty 2012; Cowey and Hardy 2006; Perez De Heredia et al. 2012). Results from animal and translational clinical studies suggest of macrophage infiltration into mammary and subcutaneous adipocytes and formation of crown-like structures around necrotic adipocyte which in turn activates the transcription factor, nuclear factor kappa-light-chain-enhancer of activated B cells **(**NF-κB) and induces pro-inflammatory mediators such as tumor necrosis factor-α (TNF- α), interleukines and cyclo-oxygenase-2 (COX-2) (Cowey and Hardy 2006; Perez De Heredia et al. 2012; Morris et al. 2011). These pro-inflammatory factors activate cytochrome P450 19 (CYP19) gene transcription yielding elevation in aromatase gene activity. (Festa et al. 2001) In addition, it has been proposed that chronic internal inflammation lends to perpetual generation of reactive oxygen and nitrogen species which in turn promotes a variety of damages ranging from mutations to post-translation modifications of proteins involved in apoptosis, DNA repair and cell cycle check points (Festa et al. 2001; Pollard 2008; Hamed et al. 2012; Hussain and Harris 2007).

Metabolic syndrome (MetS) is an amalgamation of several clinical signs and symptoms of which a minimum of three of the five risk factors, insulin resistance, hypertension, hyperlipidemia and low serum levels of HDL cholesterol, and obesity are required for diagnosis of this syndrome (Grundy et al. 2004; Grundy 2005). Remarkably, research on the association between MetS and PMBC is limited and results are not conclusive (Aghamohammadzaeh and Heagerty 2012; Bondia-Pons et al. 2012; Bjorge et al. 2010; Sinagra et al. 2002; Rosato et al. 2011; Kabat et al. 2009; Agnoli et al. 2010).

The natural history of breast cancer involves pathologically defined multi step process, starting from hyperplastic lesions to in situ and finally to invasive cancer, over a period of time (Polyak 2007; Dupont and Page 1985; Hartman et al. 2005). It is well accepted that not all women diagnosed with hyperplastic or in situ lesions subsequently are diagnosed with invasive breast cancer; nor all woman with the diagnosis of MetS eventually are diagnosed with the disease. These observations suggest that certain exogenous factors in conjunction with genetic predisposition can alter host susceptibility to carcinogenesis. In view of these observations, we conducted a retrospective study with the objective of estimating the association between MetS and PMBC; in addition, we evaluated the potential association of variants (SNPs) of eight genes which have been implicated in harboring susceptibility to adiposity, inflammation and cell proliferation (Frayling et al. 2007; Kakamani et al. 2011; Hunter et al. 2007; Dossus et al. 2008; Langsenlehner et al. 2006; Zhang et al. 2012; Healey et al. 2011; Dossus et al. 2010; Stacey et al. 2007; Andreasen et al. 2008; Rebbeck et al. 2009; Easton et al. 2007; Brasky et al. 2011).

## Materials and methods

### Study design

We implemented a prospective-specimen-collection, retrospective-blinded-evaluation (PRoBE) design approach (Pepe et al. 2008). We benefited from the MyCode prospective cohort biobanking project where blood samples are collected and procured from the primary care patient population across 31 counties within Geisinger Health System (GHS) service catchments. The banked samples are representative of the primary care patient population at GHS because of the high accrual rate of 89% of patients approached. At the time of collection, blood samples are processed according to the standard protocol, serum and DNA are then aliquoted into freezer vials, and managed by a sample tracking software FreezerWorks (Dataworks Development, Inc. Mountlake Terrace, WA) before banking in the designated freezers. All samples can be linked to various electronic databases such as Clinical Decision Information System (CDIS). The MyCode project is in full compliance with the U.S. Congress Health Insurance Portability and Accountability Act (HIPAA) of 1996 and has the approval of the Institutional Review Board.

### Case definition and identification

We defined cases as women with the diagnosis of breast cancer between January 1, 2001 and December 31, 2010. Cases were identified using the ICD-9 coding system (174.x). The MyCode database was linked to medical record numbers and subsequently to the electronic health records (EHR). Women whose diagnoses pre-dated 1/1/2001, women with diagnosis of malignancies of other organs sites except for squamous and/or basal cell carcinoma were excluded, women with medical conditions that required chronic intake of steroids and women younger than age 40 or older than 79 years were excluded.

### Control selection

Members of the cohort with no history of breast or other organ site malignancies or chronic prescription of steroids comprised the control group. We applied a ratio of one case to four controls, matched by age (± 5 years) and year of entry into the cohort. Date of blood donation to the MyCode prospective biobanking was considered the entry point for each person into the cohort.

### Data elements

Demographic and clinical data were retrieved from CDIS, an enterprise-wide data warehouse. Data were downloaded into the databases that were created for the purpose of this study.

### Data quality control and assurance

We developed a standard operational procedure for manual review of data from EHR. One of the study personnel with training in medical abstraction reviewed the EHRs over a period of nine months. The validity of electronically downloaded data was evaluated against the manually reviewed and retrieved data (Feng et al. 2013).

### Definition of metabolic syndrome

We used the World Health Organization (WHO) criteria of 1999 to classify women with or without MetS.^16^ The WHO criteria require presence of three clinically diagnosed symptoms, the diagnosis of insulin resistance in combination with two other symptoms (Table 1). Women with clinical documentation of type II diabetes, or impaired fasting glucose or impaired glucose tolerance and any two of the symptoms listed in Table 1 then were categorized into the MetS group. Height and weight data were collected from the first encounter with the health care system until the date of diagnosis of breast cancer. For each woman, we calculated her average value of weight and height that were measured across all clinical visits. The average values then were applied to calculate body mass index (BMI).

**Table 1:** **Clinical criteria for metabolic syndrome established by World Health Organization**

Clinical diagnosis	Indications
1. Insulin Resistance	Adult onset/type II diabetes or
	Impaired fasting glucose or
	Impaired glucose tolerance or
2. Hypertension	Antihypertensive medication or
	Urinary excretion rate of ≥ 20μg/min or
	Albumin:Creatinine ratio ≥ 30 mg/g
3. Hyperlipidemia	Serum triglyceride ≥ 150 mg/dl
4. HDL cholesterol level	Serum level < 39 mg/dl
5. Obesity	Body Mass Index > 30 kg/m^2^ or
	Waist:Hip ratio >0.85

### Selection of SNPs

In selecting the genes and their SNPs we reviewed findings from GWAS and other independent studies and applied minor allele frequency filtering approach and function prediction method to select a total of 64 SNPs of eight genes (Frayling et al. 2007; Kakamani et al. 2011; Hunter et al. 2007; Dossus et al. 2008; Langsenlehner et al. 2006; Zhang et al. 2012; Healey et al. 2011;Dossus et al. 2010; Stacey et al. 2007; Andreasen et al. 2008; Rebbeck et al. 2009; Easton et al. 2007; Brasky et al. 2011) (Table 2).

**Table 2:** **List of the genes and their variants (SNPs) evaluated**

Gene	Chromosomal location	SNPs
FGGR2	10q26	rs1219648, rs11200014, rs2981579
COX-2/PTGS2	1q25.2-25.3	rs2745559, rs689470, rs689466,
		rs2206593, rs5277, rs12042763,
		rs2383529
FTO	16q12.2	rs9939609, rs1861868, rs1477196
GHRL	3q26.3	rs171336, rs171407, rs35684,
		rs4684677, rs2075356, rs696217,
		rs27647, rs3755777, rs27498, rs10490815
GHSR	3q26.2	rs2948694, rs2922126
IL6	7p21.0	rs4552807 rs6969502
		rs6952003 rs10156056 rs7776857
		rs7801617 rs7805828 rs12700386
		rs1800795 rs2069840 rs2069861
		rs10242595 rs11766273
MAP3K1	5q11.2	rs889312
ESRα	6q25.1	rs2046210, rs12662670, rs3020314

### Laboratory analysis

Banked samples were retrieved and were sent to the core laboratory for analysis. All samples were marked with the study unique identifiers and the laboratory personnel and the collaborating investigators remained masked to the status of samples.

### DNA isolation

DNA was extracted from EDTA-anticoagulated whole blood using QIAsymphony SP Robot with Qiagen QIAsymphony DNA Midi Kit (Qiagen, Valencia, California) according to the manufacturer’s protocol. Quantification of extracted DNA was performed using a NanoDrop ND-1000 spectrophotometer (NanoDrop Technologies, Wilmington, Delaware).

### Genotype analysis

Single nucleotide polymorphism genotyping was performed on TaqMan® OpenArray System with assay kit (64 assay format) and Genotyping Master Mix purchased from Life Technologies (Life Technologies, Foster City, California), according to the manufacturer’s protocol. Briefly, 10 ul of each DNA samples (containing 10 ng of DNA, 5 μL of TaqMan Genotyping Master Mix, 0.25 μL of 40x assay mix, and water) plated in 384 well plate were loaded on OpenArray assay slide with Life Technologies OpenArray® AccuFill™ System (Life Technologies, Foster City, California) then performed PCR on GeneAmp PCR System 9700 (Life Technologies, Foster City, California) as follows: 93°C for 10 minutes followed by 50 cycles at 95°C for 45 seconds, 94°C for 13 seconds, and 53°C for 2 minutes 14 seconds. The post-PCR OppenArray assay slides were then scanned with OpenArray scanner and analyzed using TaqMan genotyper Software v1.3 (Life Technologies, Foster City, California). We took a two-step quality control measure to remove poor quality genotype data. First, 10% samples were replicated to test the concordance and reliability of the genotyping result. We excluded discordant SNPs. This step was followed by excluding SNPs with a recall rate of < 85% for genotyping; this step was followed by manual recall for the remaining SNPs. A total of 40 SNPs passed the two-step quality control requirement.

### Linkage disequilibrium and haplotype analysis

The observed frequencies for all selected SNPs in our sample were compared with and were in agreement with the Hardy-Weinberg-Equilibrium. We then evaluated the linkage disequilibrium structure of the SNPs in our sample using the Gabriel algorithm (Gabriel et al. 2002). (HaploView 4.0 Day Lab, Cambridge, MA). This step is followed by reconstruction of the haplotypes to evaluate the interaction between SNPs. We conducted haplotype analysis using haplo-stats Version 1.4.0 (Sinnwell, JP and Schaid DJ, built in R, version 2.7.1). In this package the maximum likelihood estimate of a haplotype probability is calculated using the EM algorithm, and used to determine possible haplotypes.

### Statistical analysis

Distributions of demographic and clinico-pathology variables between cases and controls were evaluated using non-parametric and parametric statistics. In developing the multivariate logistic regression model to determine the variables that were associated with the risk of PMBC, we first estimated the individual effect of each variable and their interactions with the outcome of interest, breast cancer. Variables with a P-value ≤ 0.10 were considered as the candidate variables. Interactions between variables also were tested at P-value ≤ .05. The final model included five candidate variables (age, smoking status, alcohol consumption status, family history of breast cancer, MetS and use of hormone replacement therapy (HRT) and the interaction between MetS and HRT. In our next analysis, we restricted the reference group to controls with no history of exposure to HRT or smoking and no clinical documentation of MetS The final model included age, family history of breast cancer, HRT, MetS and the interaction between HRT and MetS. The estimated risk of PMBC was not significantly different from our first approach where all controls were inclusive. Therefore, we use this reference group to estimate the relative risk contributions of genetic polymorphism to PMBC in presence of clinico-demographic risk factors. For each SNP, testing each SNP individually for its association with PMBC, we used the Cockerham genetic model additive coding scheme and dominant coding scheme (Cordell 2002). For the additive coding approach, we assigned the zero, one or two to each SNP genotype according to the number of copies of minor alleles. For the dominant coding scheme, we assigned the value of one for rare homozygozity and zero for the alternative homozygotes. The SNPs which showed significant association by either coding scheme, were selected (P-value < 0.1). The final multivariable model was restricted to the dominant coding scheme and was adjusted by age, family history of breast cancer, HRT, MetS and the interaction between HRT and MetS. Finally, we evaluated the risk prediction ability of the final model by plotting the receiver operating characteristic (ROC) curves and calculated area under the curve (AUC), which was equivalent to c-statistics, and reported for each model.

### Ethics

This study was approved the Institutional Review Board and is in full compliance with the U.S. Congress Health Insurance Portability and Accountability Act (HIPAA) of 1996.

## Results

**Figure 1:**
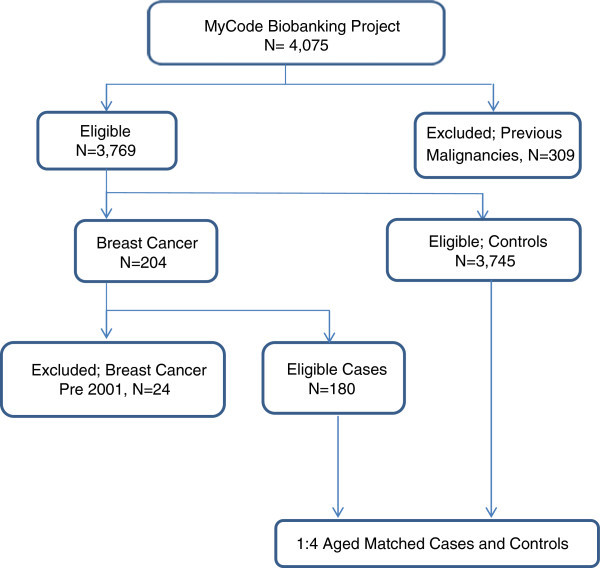
**Selection of cases and controls from the MyCode Biobanking Project.**

We identified a total of 4,075 women between ages of 40 and 79 years from the MyCode database. (Figure 1) A total of 309 women were excluded because of the history of malignancies of organ sites other than breast and/or auto-immune disorders that required chronic intake of steroids. We then conducted a search using the ICD-9 coding system for breast cancer (174.x) to identify women with the diagnosis of breast cancer in this cohort. A total of 204 women were identified of whom 24 did not meet the eligibility criteria because their diagnoses pre-dated January 1, 2001. Therefore, a total of 180 cases and 732 controls contributed to this study. The clinico-demographic characteristics of the cases and controls are presented in Table 3. Cases with the mean age of (63.1 ± 9.0) years were two years younger than controls (65.4 ±7.8). We did not detect a statistically significant difference in the mean BMI between cases (32.34 ± 7.89) and controls (32.16 ± 7.74); however, the proportion of cases (n = 49, 27.22%) who met the three criteria for MetS was significantly higher than controls (n = 24, 3.10%) (P-value < .001). The proportion of cases (n = 64, 64.44%) with medical documentation of HRT was about 1.8 fold of the control-patients (n = 280, 36.22%) (P-value < .001). Finally, twice as many cases (n = 22, 12.22%) as controls (n = 42, 5.43%) had medical documentation of first and/or second degree family history of breast cancer. Findings from the multivariate logistic regression of clinico-demographic characteristics are presented in Table 4. Advanced age was protective against PMBC (OR = 0.96, 95% CI 0.94-0.98, P-value = .001). While, family history of breast cancer (OR = 2.48, 95% CI 1.37-4.47, P-value = .003), HRT (OR = 3.13; 95% CI 2.11-4.64. P-value < .001) and MetS (OR = 12.46, 95% CI 5.62-27.65, P-value < .001) were also associated with increased risk of PMBC. Interestingly, our analysis yielded an interaction between MetS and HRT. The risk of PMBC was more the 30 fold higher for women with MetS and exposure to HRT relative to those without either (OR = 31.90, 95%CI 14.6-69.63, P-value < .001).

**Table 3:** **Clinico-demographic characteristics of case-patients and controls**

Variable	Case-patients	Controls	P-value
	N = 180 (%)	N = 773 (%)	
**Age at entry into the cohort (years)**	63.1 (± 9.00)	65.4 (± 7.80)	.001
**Body Mass Index (kg/m** ^**2**^ **)**	32.34 (±7.89)	32.16 (± 7.74)	.77
**Metabolic Syndrome**			< .001
Yes	49 (27.22)	24 (3.1)	
No	131 (72.78)	749 (96.9)	
**Smoking Status**			.06
Ever Smoked	47 (26.11)	259 (33.51)	
Never Smoked	133 (73.89)	514 (66.49)	
**Alcohol Consumption**			.69
Yes	76 (42.22)	314 (40.62)	
Never	104 (57.78)	459 (59.38)	
**Hormonal Replacement Therapy**			< .001
Yes, ever used	116 (64.44)	280 (36.22)	
No, never used	64 (35.56)	493 (63.78)	
**Family History of Breast Cancer**			.001
**(first or second degree)**			
Yes	22 (12.22)	42 (5.43)	
No	158 (87.78)	731 (94.57)	

**Table 4:** **Adjusted estimated risk of post-menopausal breast cancer: Multivariate logistic regression model; C-statistic = 0.76**

Risk factor	Cases	Controls	OR (95% CI)^1^	P-value
**Age**	180	773	0.96 (.94-.98)	.001
**First Degree Family History of Breast Cancer**				.003
Yes	22	42	1.00	
No	158	731	2.48 (1.37-4.47)	
**Smoking**				0.06
Yes	47	259	1.00	
No	133	514	0.68 (0.45-1.02)	
**HRT**				< 0.001
Yes	116	180	1.00	
No	64	493	3.13 (2.11-4.64)	
**Metabolic Syndrome**				< 0.001
Yes	49	24	1.00	
No	131	749	12.46 (5.62-27.65)	
**Metabolic Syndrome and HRT**				<.001
Yes	33	10	1.0	
No	147	763	31.90 (14.6-69.63)	

^1^OR (95% CI): Odds Ratio (95% Confidence Interval).

Frequency distributions of SNPs of the eight genes stratified by disease status are presented in Table 5. Frequency differences of one polymorphism in the GHSR gene (rs2922126), one polymorphism in IL6 gene (rs1800795) and one polymorphism in the MAP3K1 gene (rs889312) between cases and controls reached the level of statistical significance. For the gene GHSR (rs2922126), the proportion of cases (n = 158, 95.15%) with the dominant allele (T/T and A/T) was higher than the controls (n = 597, 88.18%). Similarly, prevalence of the dominant allele (C/C and G/C) of the gene IL6 (rs1800795) was higher for cases (n = 144, 87.8%) compared with the controls (n = 569, 81.4%). Finally, for the gene MAP3K1 (rs889312), analysis of our data yielded cases (n = 23, 13.86%) with a higher prevalence of recessive allele (C/C) compared with the controls (n = 41, 5.69%) (Table 5).

**Table 5:** **Frequency distribution of SNPs between cases and controls using the dominant coding scheme**

Gene, chromosomal location	SNP	Genotype	Controls	Cases	P-value
			N (%)	N (%)	
**FTO, 16 q12.2**	rs9939609	*A/A and A/T*	513 (71.35)	122 (72.62)	0.74
		*T/T*	206 (28.65)	46 (27.38)	
	rs1861868	*C/C and C/T*	526 (78.39)	123 (76.88)	0.68
		*T/T*	145 (21.61)	37 (23.13)	
	rs1477196	*G/G and A/G*	582 (82.32)	135 (82.82)	0.88
		*A/A*	125 (17.68)	28 (17.18)	
**PTGS2, 1q25.2-25.3**	rs2745559	*C/C and A/C*	632 (95.04)	157 (96.91)	0.31
		*A/A*	33 (4.96)	5 (3.09)	
	rs689466	*T/T and C/T*	483 (69.90)	112 (69.14)	0.85
		*C/C*	208 (30.10)	50 (30.86)	
	rs689470	*G/G and A/G*	617 (86.90)	153 (90.53)	0.20
		*A/A*	93 (13.10)	16 (9.47)	
	Rs2206593	*G/G and A/G*	602 (83.50)	150 (86.71)	0.30
		*A/A*	119 (16.50)	23 (13.29)	
	rs5277	C/C and C/G	656 (97.33)	155 (98.73)	0.30
		*G/G*	18 (2.67)	2 (1.27)	
	rs12042763	*G/G and G/T*	565 (87.73)	134 (86.45)	0.67
		*T/T*	79 (12.27)	21 (13.55)	
	rs2383529	*A/A and A/G*	611(94.58)	150 (96.15)	0.42
		*G/G*	35 (5.42)	6 (3.85)	
**FGGR2, 10q26**	rs1219648	*A/A and A/G*	571 (85.61)	134 (84.28)	0.67
		*G/G*	96 (14.39)	25 (15.72)	
	rs11200014	*G/G and A/G*	608 (87.11)	131 (82.39)	0.12
		*A/A*	90 (12.89)	28 (17.61)	
	rs2981582	*G/G and A/G*	581 (85.69)	128 (81.53)	0.19
		*A/A*	97 (14.31)	29 (18.47)	
**GHRL, 3q26.3**	rs171336	*G/G and G/T*	617 (87.64)	148 (90.24)	0.35
		*T/T*	87 (12.36)	16 (9.76)	
	rs171407	*A/A and A/G*	554 (80.06)	128 (83.12)	0.39
		*G/G*	138 (19.94)	26 (16.88)	
	rs35684	*A/A and A/G*	663 (92.73)	159 (95.21)	*0.25*
		*G/G*	52 (7.27)	8 (4.79)	
	rs4684677	*T/T and A/T*	699 (99.86)	163 (100.00)	0.63
		*A/A*	1 (0.14)	0 (0.00)	
	rs2075356	*T/T and C/T*	629 (87.48)	148 (91.93)	0.11
		*T/T*	90 (12.52)	13 (8.07)	
	rs27647	*T/T and C/T*	572 (82.18)	142 (87.65)	0.09
		*C/C*	124 (17.82)	20 (12.35)	
	rs3755777	*C/C and C/G*	641 (94.40)	152 (94.41)	0.99
		*G/G*	38 (5.60)	9 (5.59)	
	rs27498	*G/G and A/G*	604 (87.03)	148 (88.10)	0.71
		*A/A*	90 (12.97)	20 (11.90)	
	rs10490815	*T/T and C/T*	606 (93.09)	140 (92.72)	0.87
		*C/C*	45 (6.91)	11 (7.28)	
**GHSR**, **3q26.2**	rs2948694	*A/A and A/G*	708 (98.33)	163 (98.79)	0.67
		*G/G*	12 (1.67)	2 (1.21)	
	**rs2922126**	***T/T and A/T***	**597 (88.18)**	**158 (95.18)**	**0.008** ^**1**^
		***A/A***	**80 (11.82)**	**8 (4.82)**	
**IL6**, **7p21.0**	rs4552807	*T/T and A/T*	527 (76.16)	128 (75.74)	0.91
		*A/A*	165 (23.84)	41 (24.26)	
	rs6969502	*G/G and A/G*	674 (95.33)	158 (94.61)	0.70
		*A/A*	33 (4.67)	9 (5.39)	
	rs6952003	*T/T and A/T*	629 (94.44)	147 (94.23)	0.92
		*A/A*	37 (5.56)	9 (5.77)	
	rs10156056	*G/G and C/G*	655 (98.05)	153 (98.71)	0.58
		*C/C*	13 (1.95)	2 (1.29)	
	rs7776857	*T/T and G/T*	637 (88.23)	150 (92.02)	0.16
		*G/G*	85 (11.77)	13 (7.98)	
	rs7801617	*G/G and A/G*	631 (88.50)	158 (93.49)	0.06
		*A/A*	82 (11.50)	11 (6.51)	
	rs7805828	*A/A and A/G*	606 (87.57)	133 (83.13)	0.13
		*G/G*	86 (12.43)	27 (16.88)	
	rs12700386	*C/C and C/G*	695 (96.80)	161 (96.99)	0.90
		*G/G*	23 (3.20)	5 (3.01)	
	**rs1800795**	***C/C and G/C***	**569 (81.40)**	**144 (87.80)**	**0.05** ^1^
		***G/G***	**130 (18.60)**	**20 (12.20)**	
	rs2069840	*C/C and C/G*	633 (90.56)	151 (89.88)	0.79
		*G/G*	66 (9.44)	17 (10.12)	
	rs10242595	*G/G and A/G*	613 (89.75)	143 (91.08)	0.62
		*A/A*	70 (10.25)	14 (8.92)	
	rs11766273	*G/G and A/G*	691(98.29)	159 (100.00)	0.097
		*A/A*	12 (1.71)	0 (0.00)	
**MAP3K1**, **5q11.2**	**rs889312**	***A/A and A/C***	**679 (94.31)**	**143 (86.14)**	**< 0.001** ^**1**^
		***C/C***	**41 (5.69)**	**23 (13.86)**	
**ESR1**, **6q25.1**	rs2046210	*G/G and A/G*	573 (83.77)	139 (84.24)	0.88
		*A/A*	111 (16.23)	26 (15.76)	
	rs12662670	*T/T and G/T*	631 (92.25)	152 (95.00)	0.23
		*G/G*	53 (7.75)	8 (5.00)	
	rs3020314	*T/T and C/T*	564 (80.34)	134 (83.75)	0.32
		*C/C*	138 (19.66)	26 (16.25)	

^1^Genotype distributions between cases and controls were statistically significant.

Results from our final multivariate risk estimation analysis for SNPs combined with the clinico-demographic characteristics are presented in Table 6. One SNP (rs 2922126) in the GHSR showed a protective effect against PMBC among homozygotes for the minor allele (A/A) (OR = 0.4, 95% CI 0.18-.89, P-value = .02); while, homozygosity for the minor allele (C/C) of the SNP (rs889312) of the gene MAP3K1 was associated with the risk of PMBC (OR = 2.41, 95% CI 1.25-4.62, P-value = .008). We did not detected significant interactions between SNPs or between the SNPs and the clinico-demographic risk factors. Advanced age continued to be protective against PMBC (OR = 0.98, 95% CI 0.95-0.99, P-value = .02). While, family history of breast cancer (OR = 2.22, 95% CI 1.14-4.43. P = .02), HRT (OR = 3.35; 95% CI 2.15-5.21, P < .001), and MetS (OR = 14.83, 95% CI 5.63-39.08, P < .001) and interaction between HRT and MetS (OR = 39.38, 95% CI 15.71-98.70, P < .001) remained statistically significant risk factors for PMBC.

**Table 6:** **Adjusted estimated risk of post-menopausal breast cancer relative to the reference group, defined as women with no history of exposure to hormonal replacement therapy (HRT) and absence of medical documentation of clinical signs of metabolic syndrome (MetS); C-Statistic = 0.77**

Risk factors	Cases	ControlsW	OR (95% CI)^1^	P-value
	N = 146	N = 613		
**GHSR (rs2922126)**				.02
T/T and A/T	137	532	1.00	
A/A	9	81	.4 (.18-.89)	
**MAP3K1**				
**(rs889312 )**				
A/A and A/C	125	573	1.00	.008
C/C	21	40	2.41 (1.25-4.63)	
**Age**	146	613	.98 (.95-.99)	.02
**Family History of Breast Cancer**				.02
Yes	17	34	2.22 (1.14-4.43)	
No	129	579		
**Risk Factors**				
Reference Group	37	384	1.00	< 0.001
HRT	70	213	3.35 (2.15-5.21)	< 0.001
MetS	12	9	14.83 (5.63-39.08)	< 0.001
HRT and MetS	27	7	39.38 (15.71-98.70)	

^1^OR (95% CI) denotes Odds Ratio (95% Confidence Interval).

## Discussion

Findings from the present study further support results from previous studies that metabolic syndrome (MetS) increases the risk of postmenopausal breast cancer (PMBC) (Bjorge et al. 2010; Kabat et al. 2009; Agnoli et al. 2010; Esposito et al. 2013). We did not find an association between obesity, as measured by BMI and the risk of PMBC. In this study, we calculated BMI by taking the average of height and weight of data collected across clinical encounters, beginning with the first encounter with the system until the date of breast cancer diagnosis for all cases and their age-matched controls. Although, BMI adjusts for height, it neither adjusts for body frame size nor muscle mass. Also, it may be that insulin resistance rather than excess body weight, although highly correlated, hold the underlying biological reason for the observed increase risk of PMBC in women diagnosed with MetS. In this study, we applied the WHO criterion which recognizes the diagnosis of insulin resistance as the main symptom of MetS (Grundy et al. 2004). Gunter et al. reported a more than 2-fold increase in the risk of PMBC with fasting serum levels of insulin which was independent of BMI and other established breast cancer risk factors (Gunter et al. 2009). The complex pathophysiology of hyperinsulinemia, i.e. increased serum level of insulin-like growth factor-1 (IGF-1) and leptin and its association with the risk of PMBC has been evaluated previously and discussed extensively (Braun et al. 2011; Vatten et al. 2008; Irvin et al. 2005). IGF-1 and leptin released by visceral adipocytes have endocrine effects on several organs including breast. In addition, it has been suggested IGF-1 and leptin represent a molecular link between adipose and breast tissue (Ozhay and Nahta 2008). Adipocytes of stroma of breast epithelial cells release IGF-1 and leptin which provide paracrine growth stimulatory effects. It has proposed an autocrine signaling function as breast cancer are able to produce and secrete IGF-1 and leptin and express cell surface receptors for both ligands (Ozhay and Nahta 2008). Also, hyperinsulinemia has been associated with chronic internal inflammation and oxidative stress which have been suggested as risk factors for breast and other cancers (Bondia-Pons et al. 2012; Wiseman and Halliwell 1996).

Our findings yielded an exaggerated risk of PMBC in women diagnosed with MetS with exposure to HRT. It is well accepted that HRT increases the risk of hormone receptor positive breast cancer (Schairer et al. 2000; Ross et al. 2000). Our findings confirm the report by Gunter et al. suggesting hyperinsulinemia and serum levels of estradiol largely explain the association between obesity and PMBC (Gunter et al. 2009). Similarly, Rosenberg et al., have reported of poorer prognostic indicators at the initial clinical presentation of breast cancer and shorter overall survival among obese women using HRT when compared with obese non-users and normal body weight women (Rosenberg et al. 2009). We propose that the observed exaggerated risk of PMBC in our study sample most likely is due to the combination of an increased level of bioavailability of estradiol and an elevated susceptibility to PMBC secondary to MetS. The clinical implication of this interaction is important, given the high prevalence of obesity among the US population, particularly among African-American and Mexican-American women (2002).

Our findings suggested polymorphisms of GHSR (rs2922126) and MAP3K1 (rs889312) were associated with the risk of PMBC independent of clinico-demographic risk factors. Our results suggest homozygotes for minor allele of GHSR (rs2922126) carried a lower risk for PMBC relative to carriers of major alleles. Dossus et al. reported a 2-fold increase in the risk of breast cancer for homozygote carriers of the GHSR (rs2948694) but did not find a statistically significant association with GHSR (rs2922126) and risk of breast cancer (2010). The discrepancies in findings between these two studies potentially are due to multiple factors. First, our finding is based on a small sample size of relatively ethnically homogenous women. Second, women who contributed to our study on the average were ten years older. Third, the average BMI for women in our study was about 32 Kg/m^2^ compared with the average BMI of 26 Kg/m^2^ women who contributed to the EPIC (Dossus et al. 2010). Finally, in our study women were categorized by their MetS diagnostic measures, whereas in the EPIC study women were classified by their anthropometric measures and circulating levels of IGF-I. Gherlin and its receptor primarily have been implicated in growth hormone release, energy balance, food intake and long-term regulation of body weight. However, recent reports suggest of is complexity and multifarious system such as an inhibitory effect on pro-inflammatory cytokine expression (Gahete et al. 2011; Dixit et al. 2004).

We detected polymorphism of MAP3K1 (rs889312) was associated with an elevated risk of PMBC, independent of clinico-demographic risk factors. Our findings concur with previous studies suggesting polymorphism of MAP3KI (rs889312) was associated with the increased risk of hormone receptor positive breast cancer (Rebbeck et al. 2009; Easton et al. 2007). Although, we did not assess hormone receptor status of breast cancer cases in this study, it is well accepted that prevalence of hormone receptor positive subtype is the highest in post-menopausal women. MAP3K1 encodes mitogen-activated protein kinase protein that is involved in signal transduction pathway, a highly evolutionarily conserved mechanism of eukaroyotic cell regulation (Kyriakis and Avruch 2012). The multiple MAPK pathways present in all eukaroyotic cells enable cells to coordinate and integrate responses to a spectrum of stimuli ranging from sex-hormones, growth factors to inflammation induced cytokines and stress induced ligands (Kyriakis and Avruch 2012).

The main strength of our study was the availability of longitudinal body weight and height data. The median stay with our health care system is 18 years. Therefore, the availability of long-term data enabled us to estimate the mean body weight for each study participant which is a better reflection of the “true” body weight as oppose to a one-point-in-time measurement or self-reported body weight. Also, our study benefited from clinically documented signs and symptoms of MetS and medically documented use of HRT therapy over the period of stay of each study participant with the health care system. Therefore, the likelihood of recall bias was reduced in this study. Our study had its limitations. First, the relatively small sample size reduced the statistical power to adequately discern the association between SNPs of genes and MetS. Also, it prevented us from stratifying women by their breast cancer subtype. Also, our study sample was derived from a population relatively homogenous with respect to its genetic pool and life style risk factors. Never-the-less, our study further sheds light on the associate between prolonged MetS and the risk of PMBC.

In summary, findings from our study further confirm that MetS increases the risk of PMBC and argues in favor of reducing the exposure to HRT. In addition, our finding is another independent confirmation that low penetrance genes involved in the inflammatory pathway, i.e. MAP3KI gene, may have a plausible causative role in sporadic breast cancers. Given the fact that genetic constitutionality of individuals cannot be changed, at least at the present level of science and technology, our effort should be focused on reducing the risk of PMBC through life style modification.
